# The relationship between disability and parental status: a register study of the 1968 to 1970 birth cohorts

**DOI:** 10.1186/s12889-021-10371-1

**Published:** 2021-02-12

**Authors:** Fredinah Namatovu, Erling Häggström Lundevaller, Lotta Vikström

**Affiliations:** 1 Department of Epidemiology and Global Health, Umeå University, SE-901 87 Umeå, Sweden; 2 Centre for Demographic and Ageing Research (CEDAR), Umeå University, SE-901 87 Umeå, Sweden; 3 Umeå School of Business, Economics, and Statistics, Umeå University, SE-901 87 Umeå, Sweden; 4 Department of Historical, Philosophical, and Religious Studies, SE-901 87 Umeå, Sweden

**Keywords:** Child, Children, Disability, Disability benefits, Disability pension, Early retirement, Fertility, Having children, Parenthood, Parenting, Parental status

## Abstract

**Background:**

Having children is a major life course event yet some disabilities could make it biologically challenging and some others could limit access to necessary socioeconomic resources. To date, there is relatively little data on disability and parental status and our study aimed to investigate this relationship.

**Methods:**

This longitudinal cohort study was based on register data obtained from all people born in Sweden from 1968 to 1970 (*n* = 440220). We performed descriptive analyses, graphical plots, logistic regression, and Cox regression analyses.

**Results:**

Our findings from both logistic regression and Cox regression indicated that individuals that started to receive disability benefits at an early age had reduced chances of having children during the follow-up duration. Men with disabilities were less likely to have children when compared to women with disabilities and to men and women without disabilities.

**Conclusions:**

We found evidence that disability during early adulthood was associated with reduced chances of having children. Findings support policies and programmes aimed at promoting optimal health during early adulthood, as this would promote continued labour force participation, reduce early use of disability benefits, and possibly improve chances of becoming a parent.

**Supplementary Information:**

The online version contains supplementary material available at 10.1186/s12889-021-10371-1.

## Background

Many societies consider having children a major life course event [1]. In modern societies, children are shown to offer psychosocial benefits, such as the intrinsic pleasure derived from watching one’s own children grow and having someone for regular interaction [2]. Having children is also associated with improved physical health and enhanced health behaviours, such as cessation of smoking and alcohol abuse [3]. However, the decision to have or not to have children is based on an individual’s current and future circumstances [1, 4]. Several researchers have shown that fertility patterns are fundamentally affected by social, cultural, and economic factors [5, 6]. In high income economies, there are several pivotal factors that are often fulfilled before starting to have children. Such factors include being out of school, having a steady income, living independently, and having a partner [5, 6].

Having a disability can profoundly affect one’s ability to fulfil the pivotal determinants for having a first child and might lead to postponement or in some cases to childlessness. Some disabilities make it biologically impossible to have children [7], while others might limit access to the required socioeconomic resources [8]. Literature operationalises disabilities differently, some definitions include self-reported disability, disability diagnosis and the administrative definition. In this study, we use an administrative definition that considers one to have a disability if a person receives disability benefits [9–11]. In Sweden, disability benefits are part of a public social security programme that provides income support to people of working age that experience long-term health limitations in their working capacity [9–11]. Having a disability that requires exiting the work force at a young age increases the length of time spent outside of a work environment, which can jeopardise one’s health, social and economic conditions over their lifetime [12]. European data suggests that the number of young people exiting the labour market early is increasing [12]. However, there is limited research on whether disability that leads to early exit from the labour force is associated with reduced chances of having children.

From a theoretical point of view, the link between disability and parental status can be understood using Oppenheimer’s uncertainty hypothesis [13, 14] and the theory of assortative mating [15, 16]. Oppenheimer’s uncertainty hypothesis suggests that insufficient economic resources hinder marriage [13, 14]. Exiting the labour force and being on disability benefits early in life might create financial constraints and economic uncertainty. Oppenheimer also argues that assortative mating might occur when individuals with low economic resources are considered undesirable for marriage [13, 14]. Due to assortative mating on disability, people without disabilities might prefer to choose partners that do not have disabilities. Disability researchers have used the theory of assortative mating to explain why disability is linked to reduced chances of marriage and cohabitation [15–18]. Disability that occurs during early adulthood could contribute to assortative mating, creates economic constraints, makes it difficult for one to have a steady income, live independently and have a partner, which results in the postponement of parenthood or even childlessness.

Current literature suggests that people with disabilities report limited economic opportunities and high poverty rates [8, 19]. Several studies also indicate that disability is associated with low levels of partnership and high levels of single living [17, 18, 20–22]. People with disabilities also report negative societal attitudes characterised by infantilisation and being treated as asexual [22–24]. Structural barriers and limited access to fertility services are additional challenges reported in this population [25, 26]. All these factors negatively impact the chances of having children among people with disabilities.

Compared to the extensive body of research on parenthood in the general population, there is relatively little data on the parental status of people with disabilities. Available research can be divided into two subsets. One body of research examines the experiences of parenting with a disability and being a parent to a child with a disability [22–28]. Another branch of research focuses on the parenthood status of people with learning disabilities and physical disabilities [25–28]. There is still little research on the extent to which people with disabilities become parents and the association between disability and parental status.

### Aims

The aim of this study was to examine the relationship between having a disability in early adulthood and reduced chances of having children.

## Methods

### Design and study setting

In this longitudinal cohort study, we used national register data that consisted of all people that were born in Sweden (*n* = 440220) from 1968 to 1970. Individuals were followed from birth until 2010 when the youngest were 39 years old and the oldest were 42 years old. The selection of this study duration was based on data availability. All data was obtained from the Longitudinal Integration Database for Health Insurance and Labour Market Studies (LISA database). Our data consisted of information on total population, disability benefits status, number of children, sex, and year of birth. All data was anonymised by Statistics Sweden and was made available for analysis through the Swedish Initiative for Research on Microdata in Social and Medical Sciences (Umeå SIMSAM Lab) [29].

### Outcome variable

The outcome variable was whether an individual became a parent or not from age 23 up to the end of the study duration. The variable “becoming a parent” was considered binary, which indicated whether an individual had a biological child or not during the observation time. Individuals who became parents at age 13–22 years, 12% (*n* = 51092) were excluded from the from the regression analyses.

### Exposure variables

The main exposure variable to indicate disability status was “whether an individual received a disability benefit at age 20–22 years or not.” Eligibility for receipt of these benefits was confirmed through a medical examination which indicated diminished health and work capacity [9–11]. During the study duration, the disability benefits were referred to as a disability pension and were awarded to medically eligible people aged between 16 and 64 years [9–11]. We coded the individuals who received disability benefits “yes” at the first year they received the benefits; those who did not receive disability benefits during the entire study duration were coded “no”. For this study, age at first receipt of disability benefits ranged between 20 and 22 years. Those aged 20 years could have started on the benefits earlier than this, however this was the age when they were first registered in the LISA database following its establishment in 1990. The other covariate included was the year of birth, which ranged between 1968 and 1970.

### Statistical analyses

**Fig. 1:**
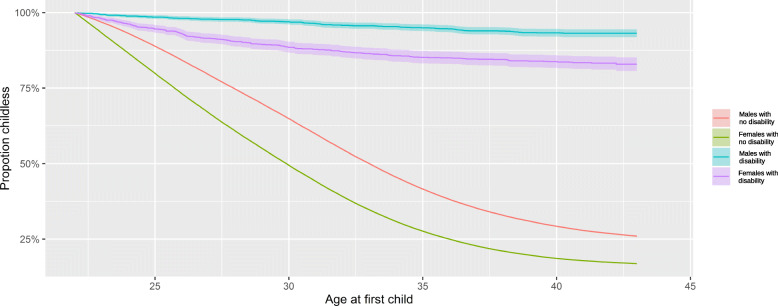
The Kaplan-Meier survival plot for age at first child

Descriptive statistics were performed using frequency tables to give an overview of the dataset. We plotted the Kaplan-Meier curve (Fig. 1) showing the proportion surviving childless at each age after 22 years of age.

Logistic regression analyses were performed for men and women separately to assess the association between having a disability at ages 20–22 and later having a child. Independent models for disability benefits and year of birth and models that simultaneously included the two variables were reported (Table 2). Regression results were presented as odds ratios (ORs) with 95% confidence intervals (CIs) and statistical significance set at *p* < 0.05.

Using Cox proportional hazard regression, we modelled having a child during the follow-up. Disability status was assessed at age 20–22 years, and age at having a first child set at 23 years and onwards to ensure the temporal order in this longitudinal data. Individuals entered the study at age 20–22 and were followed until they had their first child or until they were censored, out migrated, died, or the end of study period on 31, December 2010. We reported four models, which included independent and adjusted associations fitted for men and women separately. In the results presented in the Appendix, we repeated both the independent and adjusted logistic regression and Cox regression analyses incorporating marital status, to disentangle the effect of marriage. We further tested the interactions between disability and marital status. All analysis was performed using R software 4.0.2 for windows.

## Results

For the Swedish birth cohorts of 1968 to 1970, childlessness was more than double among people with disabilities compared to those without disabilities. For those with a disability, 13% had at least one child compared to 73% without a disability. The number of men was slightly higher than that of women (51% vs. 49%). The 1968 birth cohort had the highest proportion with disabilities (Table 1).

**Table 1:** The distribution of Swedish men and women born between 1968 and 1970 by selected demographic characteristics presented for those with or without disability at age 20–22 years (*N* = 440220)

Descriptive		No Disability (*n* = 437,367)	^a^Disability (2853)	Total (440220)
		N (%)	N (%)	N (%)
Any children	No	119,530 (27)	2488 (87)	122,018 (28)
	Yes	317,837 (73)	365 (13)	318,202 (72)
Number of children	1	64,410 (15)	139 (05)	64,549 (15)
	2+	253,427 (58)	226 (08)	253,653 (58)
Age at first child	No Child	119,530 (27)	2488 (87)	122,018 (28)
	13–22^b^	51,021 (12)	71 (02)	51,092 (12)
	23–25	57,656 (13)	88 (03)	57,744 (13)
	26–30	96,833 (22)	98 (03)	96,931 (22)
	31–35	76,196 (17)	65 (02)	76,261 (17)
	36–42	36,131 (08)	43 (02)	36,174 (09)
Sex	Men	222,989 (51)	1592 (56)	224,581 (51)
	Women	214,260 (49)	1261 (44)	215,521 (49)
Year of index birth	1968	149,709 (34)	829 (29)	150,538 (34)
	1969	142,943 (33)	927 (32)	143,870 (33)
	1970	144,715 (33)	1097 (38)	145,812 (33)

^a^Disability present at age 20–22, ^b^these were later excluded from the regression analyses

Figure 1 shows that childlessness was more common among those with disabilities. Childlessness rapidly decreased with time in the group without disabilities but remained high and barely changed in the group with disabilities. At age 30, about 80% of the women and 95% of men with disabilities were childless, with meagre fluctuations by the end of the follow-up. Corresponding figures among those without disabilities were 50% for women and about 65% for men at age 30, this further dropped to about 10% in women and over 25% in men.

### Regression results

The bivariate and multivariable logistic regression results presented in Table 2, showed that men and women that received their first disability benefits before their 23rd birthday were significantly less likely to become parents compared to those that never received disability benefits. In addition, men with disabilities were less likely to become parents compared to women with disabilities. The birth cohorts of 1969 and 1970 had lower chances of having children, compared to the birth cohort of 1968, regardless of sex.

**Table 2:** Logistic regression results on the association between receiving disability benefits at 20–22 years and having a first child at a later age

Variable	Men		Women	
	COR (95% CI)	AOR (95% CI)	COR (95% CI)	AOR (95% CI)
Disability benefits: No	1.0	1.0	1.0	1.0
At 20–22 yrs	0.02 (0.02–0.03)	0.02 (0.02–0.03)	0.04 (0.03–0.04)	0.04 (0.03–0.04)
Year of birth 1968		1.0		1.0
1969		0.97 (0.94–1.00)		0.96 (0.92–0.99)
1970		0.93 (0.90–0.96)		0.95 (0.92–0.99)

*COR* Crude Odds Ratio, *AOR* Adjusted Odds Ratio, *CI* Confidence Interval

The hazard ratio from the Cox regression presented in Table 3 also confirmed results from logistic regression, disability was significantly associated with reduced chances of having children, regardless of sex. Men with disabilities were less likely to become parents compared to women with disabilities. Again, becoming a parent was less common in younger birth cohorts compared to the birth cohort of 1968.

**Table 3:** The hazard ratio of having a first child later if you received disability benefits at 20–22 years

Variable	Men		Women	
	CHR (95% CI)	AHR (95% CI)	CHR (95% CI)	AHR (95% CI)
Disability benefits: No		1.00	1.00	1.00
at 20–22 yrs	0.06 (0.05–0.07)	0.06 (0.05–0.07)	0.11 (0.09–0.13)	0.11 (0.09–0.13)
Year of birth 1968	1.0	1.00	1.00	1.00
1969		0.98 (0.97–0.99)		0.96 (0.95–0.98)
1970		0.95 (0.94–0.97)		0.95 (0.93–0.96)

*CHR* Crude Hazard Ratio, *AOR* Adjusted Hazard Ratio, *CI* Confidence Interval

The results presented in the Appendix, also confirmed that disability was significantly associated with reduced chances of having children even after adjusting for marital status and assessing the interaction between disability and marriage. This association was confirmed in both the logistic regression and Cox regression models, Tables S1, S2, S3 and S4.

## Discussion

Our register data for the birth cohorts of 1968 to 1970 showed that having a disability during early adulthood was associated with extremely low chances of having children during the observed duration. Men with disabilities were less likely to have children compared to women with disabilities and to men and women without disabilities. This study also noted that the chances of having children were reduced for younger birth cohorts compared to the birth cohort of 1968.

Our finding that receiving disability benefits was associated with significant reduction in chances of having children support findings from previous studies that reported similar observations [20, 21]. The association between disability and low chances of having children could very well be explained by several factors including economic barriers as Oppenheimer’s theory suggests [13, 30] and by assortative mating on disability status [15, 16]. In addition, biological barriers and negative societal attitudes on parenting with a disability could also negatively influence the likelihood of becoming a parent [24, 25].

Having a disability in early adulthood was associated with a significant reduction in the chances of becoming a parent later in life. Starting to receive disability benefits during the early twenties might imply prolonged ill health and socio-economic constraints, factors that might constrain the decision to become a parent. Moreover, previous data has shown that the majority of individuals who start to receive disability benefits continue to do so on a long-term basis, with an outflow rate at just about 3 % [12].

This study also identified major sex differences in the chances of having children. The chances were much lower for men with disabilities compared to women with disabilities or to men and women without disabilities. Literature suggests that entry into a stable partnership requires a strong financial underpinning and more occupational stability for men compared to women [13, 14]. Limited male entry into partnership formation will subsequently result in low numbers of children for men. However, it is also important to note that our study population consisted of a higher proportion of men than women, implying a surplus of men on the partnership market, which further constrain the partnership chances of men with disabilities.

We noted that the odds of having children were reduced in the younger birth cohorts of 1969 and 1970 compared to people born in 1968. There are no known social economic changes during this time to explain these parental status differences by birth cohort. It is therefore possible that this association is due to random variation.

### Strengths and limitations of the study

The main strength of this study is the access to a large longitudinal dataset that gave us an opportunity to observe a total population for a long duration. Moreover, using register data ensured minimal loss to follow-up, data on the use of disability benefits and having children was available for all cohorts. In this study, we define disability status based on use of disability benefits, we consider this a reliable measure for disability status and the severity of the disability given that eligibility was based on medical evidence suggesting long-term health limitations in working capacity. Even though this study found a strong association between disability and reduced chances of having children, this should be strictly interpreted as an association and should not be used to draw causal conclusions. In addition, our findings should be interpreted within the frames of the studied type of disability. Further research using other definitions of disabilities and study designs could help clarify the observed association between disability and chances of having children. Even though our models excluded some people due to missing data, there is no reason to suspect that missing data differed by disability status.

## Conclusion

This study shows a strong association between receiving disability benefits and reduced chances of having children. Moreover, men with disabilities were at reduced chances of having children compared to women with disabilities and to men and women without disabilities. Our findings support policies and programmes aimed at promoting good health during reproductive age as this could reduce early exit from work and early use of disability benefits. Further research is needed to clarify the association between use of disability benefits and all factors related to reproductive health, marriage, and parenthood and family relationships among people with disabilities. Such evidence will help inform both public health and family policies.

## Supplementary Information

**Additional file 1: Table S1.** Odds of later having a first child for men that receive a disability benefit at 20–22 years of age in Sweden. Interaction between disability benefit and marriage. COR = Crude Odds Ratio; AOR = Adjusted Odds Ratio; CI=Confidence Interval.

**Additional file 2: Table S2.** Odds of later having a first child for women that receive a disability benefit at 20–22 years of age in Sweden. Interaction between disability benefit and marriage. COR = Crude Odds Ratio; AOR = Adjusted Odds Ratio; CI=Confidence Interval.

**Additional file 3: Table S3.** Hazard results of later having a first child for men that receive a disability benefit at 20–22 years of age in Sweden. Interaction between disability benefit and marriage. CHR = Crude Hazard Ratio; AOR = Adjusted Hazard Ratio; CI=Confidence Interval.

**Additional file 4: Table S4.** Hazard results of later having a first child for women that receive a disability benefit at 20–22 years of age in Sweden. Interaction between disability benefit and marriage. CHR = Crude Hazard Ratio; AOR = Adjusted Hazard Ratio; CI=Confidence Interval.

## Data Availability

The datasets supporting the conclusions of this article are available in the Statistics Sweden repository and can be made available to researchers in accordance with the ethical and legal restrictions regarding Swedish Public Access to Information and Secrecy Act data. Statistics Sweden is a Swedish government agency responsible for official statistics in Sweden. To request access to this data contact: information@scb.se, + 46104795000. Researchers requesting access to this data should also have obtained ethical approval from the Swedish Central Ethical Review Board, its contact information is: registrator@etikprovning.se, telephone: + 46104750800.

## Abbreviations

CI: Confidence interval
LISA: Longitudinal Integration Database for Health Insurance and Labour Market Studies
SIMSAM: Swedish Initiative for Research on Microdata in Social and Medical Science
