# Supplementary material for: The relationship between disability and parental status: a register study of the 1968 to 1970 birth cohorts
Source: BMC Public Health. 2021 Feb 12;21:343. doi: 10.1186/s12889-021-10371-1 (PMC7881455; doi:10.1186/s12889-021-10371-1)
Supplement: Supplementary file 1 — Additional file 1: Table S1. Odds of later having a first child for men that receive a disability benefit at 20–22 years of age in Sweden. Interaction between disability benefit and marriage. COR = Crude Odds Ratio; AOR = Adjusted Odds Ratio; CI=Confidence Interval. [file 12889_2021_10371_MOESM1_ESM.docx]

Table S1. Odds of later having a first child for men that receive a disability benefit at 20-22 years of age in Sweden. Interaction between disability benefit and marriage.

|  |  |  |  |  |
| --- | --- | --- | --- | --- |
|  | Model 1 | Model 2 | Model 3 | Model 5 |
| Variable | OR (P-value) | OR (P-value) | OR (P-value) | OR (P-value) |
| Disability benefits: No | 1.0 | 1.0 | 1.0 | 1.0 |
| at 20–22yrs | 0.04 (0.03–0.04) | 0.04 (0.03–0.04) | 0.04 (0.03–0.04) | 0.02 (0.01–0.03) |
| Year of birth 1968 |  | 1.0 | 1.0 | 1.0 |
| 1969 |  | 0.96 (0.92–0.99) | 0.97 (0.93–1.00) | 0.97 (0.94–1.00) |
| 1970 |  | 0.95 (0.92–0.99) | 0.93 (0.90–0.96) | 0.93 (0.90–0.96) |
| Married No |  |  | 1.0 | 1.0 |
| Yes |  |  | 8.70 (8.45–8.97) | 8.66 (8.39–8.92) |
| Disability*Married |  |  |  | 1.0 |
|  |  |  |  | 4.91 (2.92–8.39) |

COR=Crude Odds Ratio; AOR=Adjusted Odds Ratio; CI=Confidence Interval
