# Supplementary material for: The relationship between disability and parental status: a register study of the 1968 to 1970 birth cohorts
Source: BMC Public Health. 2021 Feb 12;21:343. doi: 10.1186/s12889-021-10371-1 (PMC7881455; doi:10.1186/s12889-021-10371-1)
Supplement: Supplementary file 2 — Additional file 2: Table S2. Odds of later having a first child for women that receive a disability benefit at 20–22 years of age in Sweden. Interaction between disability benefit and marriage. COR = Crude Odds Ratio; AOR = Adjusted Odds Ratio; CI=Confidence Interval. [file 12889_2021_10371_MOESM2_ESM.docx]

Table S2. Odds of later having a first child for women that receive a disability benefit at 20-22 years of age in Sweden. Interaction between disability benefit and marriage.

|  |  |  |  |  |
| --- | --- | --- | --- | --- |
|  | Model 1 | Model 2 | Model 3 | Model 5 |
| Variable | COR (95 % CI) | AOR (95 % CI) | AOR (95 % CI) | AOR (95 % CI) |
| Disability benefits: No | 1.0 | 1.0 | 1.0 | 1.0 |
| at 20–22yrs | 0.02 (0.02–0.03) | 0.04 (0.02–0.03) | 0.04 (0.03–0.05) | 0.04 (0.03–0.05) |
| Year of birth 1968 |  | 1.0 | 1.0 | 1.0 |
| 1969 |  | 0.97 (0.94–0.99) | 0.97 (0.94–1.99) | 0.97 (0.93–1.01) |
| 1970 |  | 0.91 (0.88–0.94) | 0.91 (0.88–0.94) | 0.97 (0.9 3–1.00) |
| Married No |  |  |  | 1.0 |
| Yes |  |  |  | 5.86 (5.66–6.08) |
| Disability*Married |  |  |  | 1.0 |
|  |  |  |  | 3.01 (2.03–4.51) |

COR=Crude Odds Ratio; AOR=Adjusted Odds Ratio; CI=Confidence Interval
