# Supplementary material for: The relationship between disability and parental status: a register study of the 1968 to 1970 birth cohorts
Source: BMC Public Health. 2021 Feb 12;21:343. doi: 10.1186/s12889-021-10371-1 (PMC7881455; doi:10.1186/s12889-021-10371-1)
Supplement: Supplementary file 3 — Additional file 3: Table S3. Hazard results of later having a first child for men that receive a disability benefit at 20–22 years of age in Sweden. Interaction between disability benefit and marriage. CHR = Crude Hazard Ratio; AOR = Adjusted Hazard Ratio; CI=Confidence Interval. [file 12889_2021_10371_MOESM3_ESM.docx]

Table S3. Hazard results of later having a first child for men that receive a disability benefit at 20-22 years of age in Sweden. Interaction between disability benefit and marriage.

|  |  |  |  |  |
| --- | --- | --- | --- | --- |
|  | Model 1 | Model 2 | Model 3 | Model 5 |
| Variable | CHR (95 % CI) | AHR (95 % CI) | AHR (95 % CI) | AHR (95 % CI) |
| Disability benefits: No |  | 1.00 | 1.00 | 1.00 |
| at 20–22yrs | 0.06 (0.05–0.07) | 0.06 (0.05–0.07) | 0.09 (0.07–0.10) | 0.04 (0.03–0.05) |
| Year of birth 1968 |  | 1.00 | 1.00 | 1.00 |
| 1969 |  | 0.98 (0.97–0.99) | 0.98 (0.97–0.99) | 0.98 (0.97–0.99) |
| 1970 |  | 0.95 (0.94–0.97) | 0.97 (0.96–0.98) | 0.97 (0.95–0.98) |
| Married No |  |  | 1.00 | 1.00 |
| Yes |  |  | 2.68 (2.66–2.71) | 2.68 (2.64–2.07) |
| Disability*Married |  |  |  | 11.4 (7.63–11.9) |

CHR=Crude Hazard Ratio; AOR=Adjusted Hazard Ratio; CI=Confidence Interval
